# Supplementary material for: Use of Two PGPB Strains for the Valorization of Wastewater Sludge and Formulation of a Biofertilizer for the Recovery of Quercus ilex
Source: Life (Basel). 2025 Sep 22;15(9):1490. doi: 10.3390/life15091490 (PMC12472160; doi:10.3390/life15091490)
Supplement: Supplementary file 1 [file life-15-01490-s001.zip › life-3827672-supplementary.pdf]

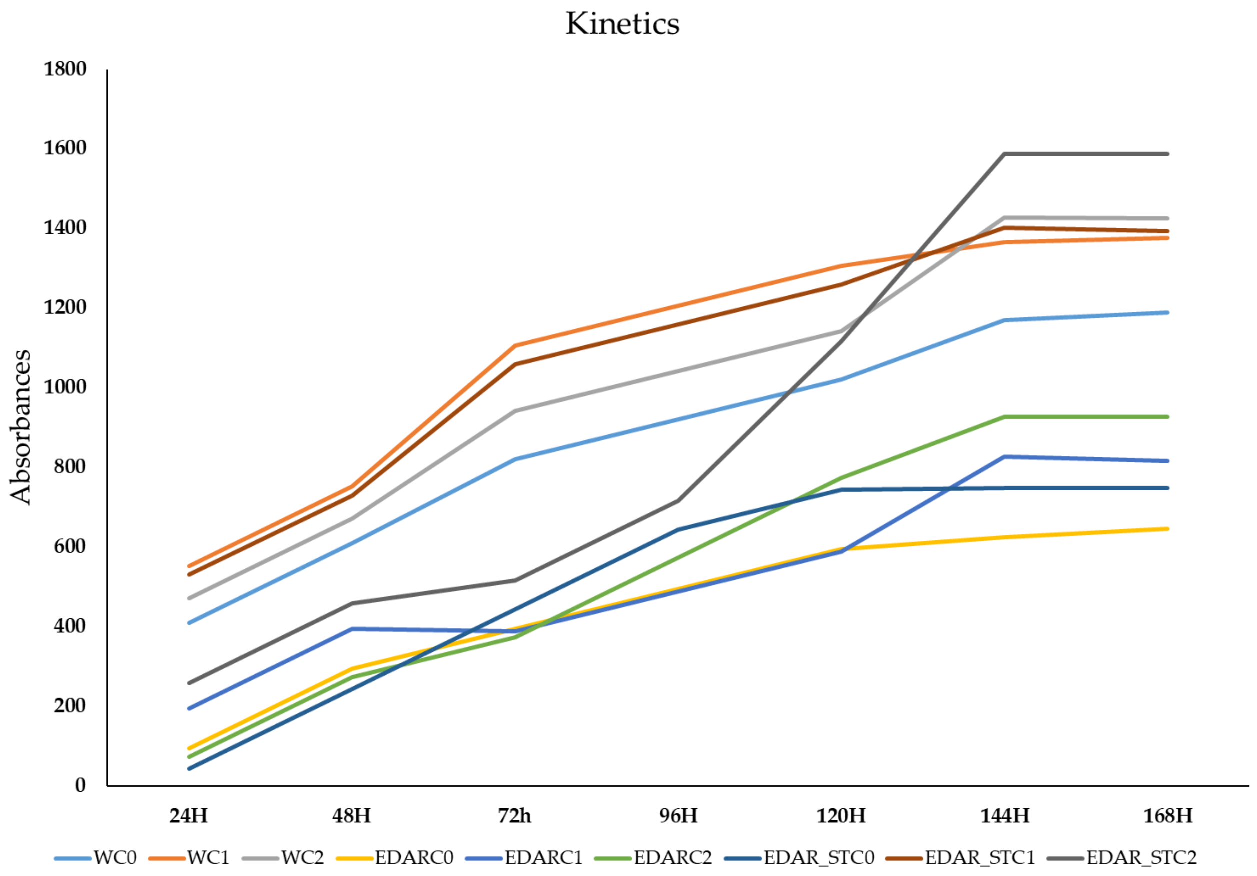

Figure S1. Kinetics of Biolog Ecoplate absorbances.

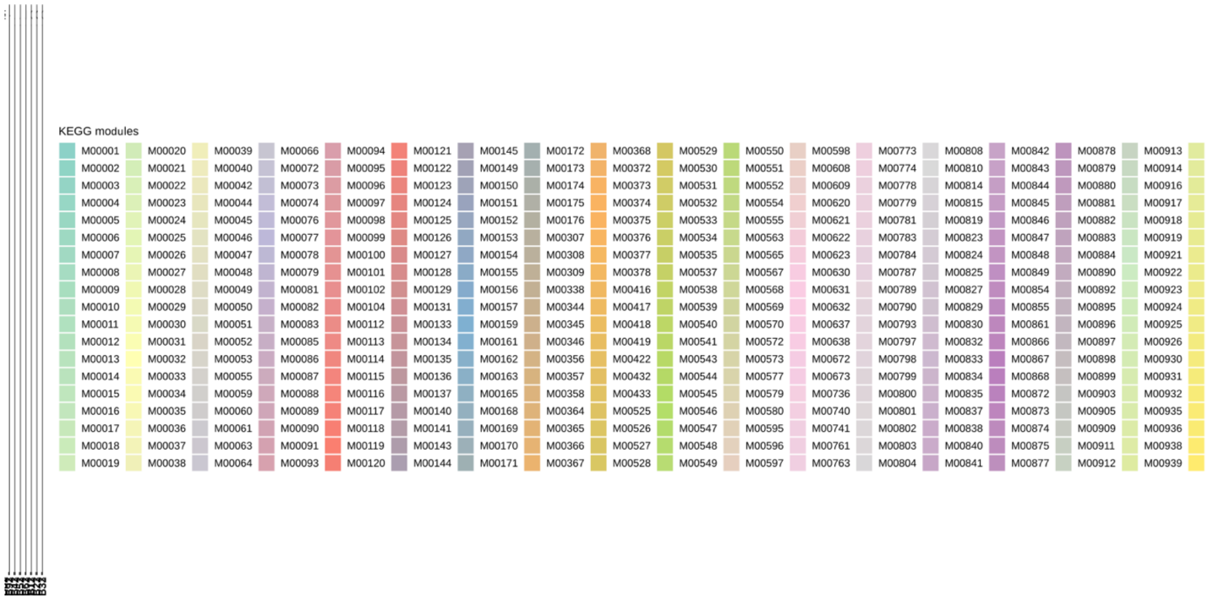

Figure S2. Functional distribution of KEGG modules predicted by PICRUSt2. Relative abundance representation for complete metabolic modules. Homogeneous profile with slight differences between treatments.

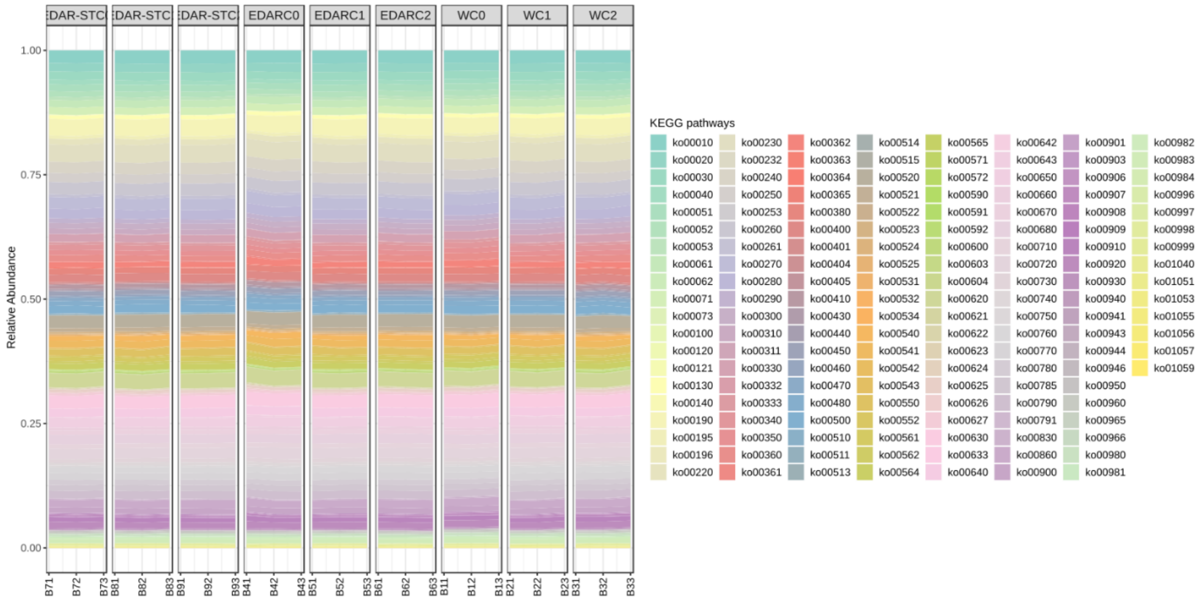

Figure S3. Functional prediction of complete KEGG pathways. Visualization of more than 100 functional pathway-level paths. Most of them have low relative variability between treatments.
